# Combination of unsaturated fatty acids and ionizing radiation on human glioma cells: cellular, biochemical and gene expression analysis

**DOI:** 10.1186/1476-511X-13-142

**Published:** 2014-09-02

**Authors:** Otilia Antal, László Hackler, Junhui Shen, Imola Mán, Katalin Hideghéty, Klára Kitajka, László G Puskás

**Affiliations:** Laboratory for Functional Genomics, Institute of Genetics, Biological Research Center of the Hungarian Academy of Sciences, Szeged, H-6726 Hungary; Avidin Ltd, Szeged, H-6726 Hungary; Tongji University School of Medicine, Shanghai, 200092 China; Department of Oncotherapy, Faculty of Medicine, University of Szeged, Szeged, H-6720 Hungary

**Keywords:** Polyunsaturated fatty acids (PUFAs), Glioma, Irradiation, Gene expression, miRNA expression

## Abstract

**Background:**

Based on previous observations a potential resort in the therapy of the particularly radioresistant glioma would be its treatment with unsaturated fatty acids (UFAs) combined with irradiation.

**Methods:**

We evaluated the effect of different UFAs (arachidonic acid (AA), docosahexaenoic acid (DHA), gamma-linolenic acid (GLA), eicosapentaenoic acid (EPA) and oleic acid (OA)) on human U87 MG glioma cell line by classical biochemical end-point assays, impedance-based, real-time cellular and holographic microscopic analysis. We further analyzed AA, DHA, and GLA at morphological, gene and miRNA expression level.

**Results:**

Corresponding to LDH-, MTS assays and real-time cytoxicity profiles AA, DHA, and GLA enhanced the radio sensitivity of glioma cells. The collective application of polyunsaturated fatty acids (PUFAs) and irradiation significantly changed the expression of *EGR1*, *TNF-α*, *NOTCH1*, *c-MYC*, *TP53*, *HMOX1*, *AKR1C1*, *NQO1*, while up-regulation of *GADD45A*, *EGR1*, *GRP78*, *DDIT3*, *c-MYC*, *FOSL1* were recorded both in response to PUFA treatment or irradiation alone. Among the analyzed miRNAs miR-146 and miR-181a were induced by DHA treatment. Overexpression of miR-146 was also detected by combined treatment of GLA and irradiation.

**Conclusions:**

Because PUFAs increased the radio responsiveness of glioma cells as assessed by biochemical and cellular assays, they might increase the therapeutic efficacy of radiation in treatment of gliomas. We demonstrated that treatment with DHA, AA and GLA as adjunct to irradiation up-regulated the expression of oxidative-stress and endoplasmic reticulum stress related genes, and affected *NOTCH1* expression, which could explain their additive effects.

**Electronic supplementary material:**

The online version of this article (doi:10.1186/1476-511X-13-142) contains supplementary material, which is available to authorized users.

## Background

Glioblastoma is among the most lethal tumor types, the median survival time of patients following diagnosis is less than two years. Glioma is the most common malignancy of the central nervous system in adults [1]. Designing new therapeutic methods for treating glioblastoma remains an important task of the research community, due to its high resistance to irradiation and chemotherapy. Currently, the primary method for treatment of glioblastoma is surgical resection in combination with radiotherapy and in several cases chemotherapy [2–4]. Unfortunately, due to the radioresistance of glioma-initiating cells (cells with cancer stem cell characteristics), the rate of recurrence is extremely high [5].

Previous studies have pointed out that PUFAs are useful as adjuncts in cancer treatment beside irradiation and chemotherapy, both *in vitro* and *in vivo* [6–11]. Arachidonic acid (AA, 20:4n-6), docosahexaenoic acid (DHA, 22:6n-3), gamma-linolenic acid (GLA, 18:3n-6) and eicosapentaenoic acid (EPA, 20:5n-3) *per se* induced apoptosis of cancerous cells [6–9]. According to studies on glioma spheroids grown on collagen gels and on several glioma cell lines (C6, U373, U87 MG) GLA treatment was cytotoxic, while it did not influence normal cells [11]. *In vivo,* GLA treatment did not influence normal brain tissue and it caused the regression of glioblastomas in human patients, without detectable side-effects or acute inflammatory response [10–12]. In a pilot study, GLA was applied as a therapeutic agent after surgery; it was administered by intracranial infusion, and it was found that it is neuroprotective with minimal side-effects. Experiments performed on rat and human brains suggest that GLA infusion through the intraparenchymal route is an effective method, it could appreciably expand the life-expectancy of glioblastoma patients, it could even double the survival period from 2 to 4 years [11, 13, 14].

Leary et al. found that GLA acts more selectively on human oesophageal carcinoma cells, than AA and EPA [15]. GLA treatment diminished anti-oxidant levels in tumor cells which may be beneficial, because anti-oxidants inhibit the apoptotic effect of GLA on cancer cells. At the same time, the genotoxic and cytotoxic effect of chemotherapeutics and radiation was attenuated by GLA treatment [11].

In a clinical study, EPA and DHA supplementation was found to be beneficial in lung cancer treatment [16]. ω-3 PUFAs facilitated the uptake of chemotherapeutic drugs, *in vitro* enhanced their cytotoxic effect. EPA and DHA supplementation associated with the administration of several chemotherapeutics diminished tumor size and alleviated side effects [17].

It was described that PUFAs can increase the cytotoxicity of numerous chemotherapeutics *in vitro,* in brain, lung, breast, sarcoma, lymphocytic, colon human cell cultures [17–20]. PUFAs also inhibited cachexia in animal models; suppressed neoplastic transformation; inhibited angiogenesis and metastasis [21].

One possibility to achieve a more intense antitumor effect would be the combination of fatty acids with radiotherapy, which was proven to be beneficial both *in vivo* and *in vitro.* DHA enhanced the responsiveness of mammary tumors to ionizing radiation, and it did not influence the radio-sensitivity of normal tissue [22]. The exact mechanism by which DHA in combination with radiotherapy exerts its specific effect on tumors is yet unknown, but lipid peroxidation can be a contributing factor [19, 22]. The same hypothesis could also stand for GLA treatment. Furthermore, GLA treatment protected mice bone marrow cells from irradiation-induced DNA damage [Das, 2007 = reference 11]. GLA could also sensitize astrocytoma to radiotherapy, while on normal cells it had a cytoprotective effect [10, 11]. GLA, AA and EPA had a synergistic effect with irradiation on C6 glioma cells, enhancing the rate of apoptosis [13].

During the present study we investigated the interaction between UFAs and irradiation on human U87 MG glioma cell line, by biophysical and biochemical assays, holographic imaging and quantitative PCR based assays.

The molecular pathways that are affected in glioblastoma, the genetic interaction network through which UFA treatment and irradiation can selectively kill cancer cells is still undetermined. The assessment of genes and miRNAs with altered expression due to PUFA treatment and irradiation can be the foundation of improved and more effective therapies.

## Results

**Figure 1:**
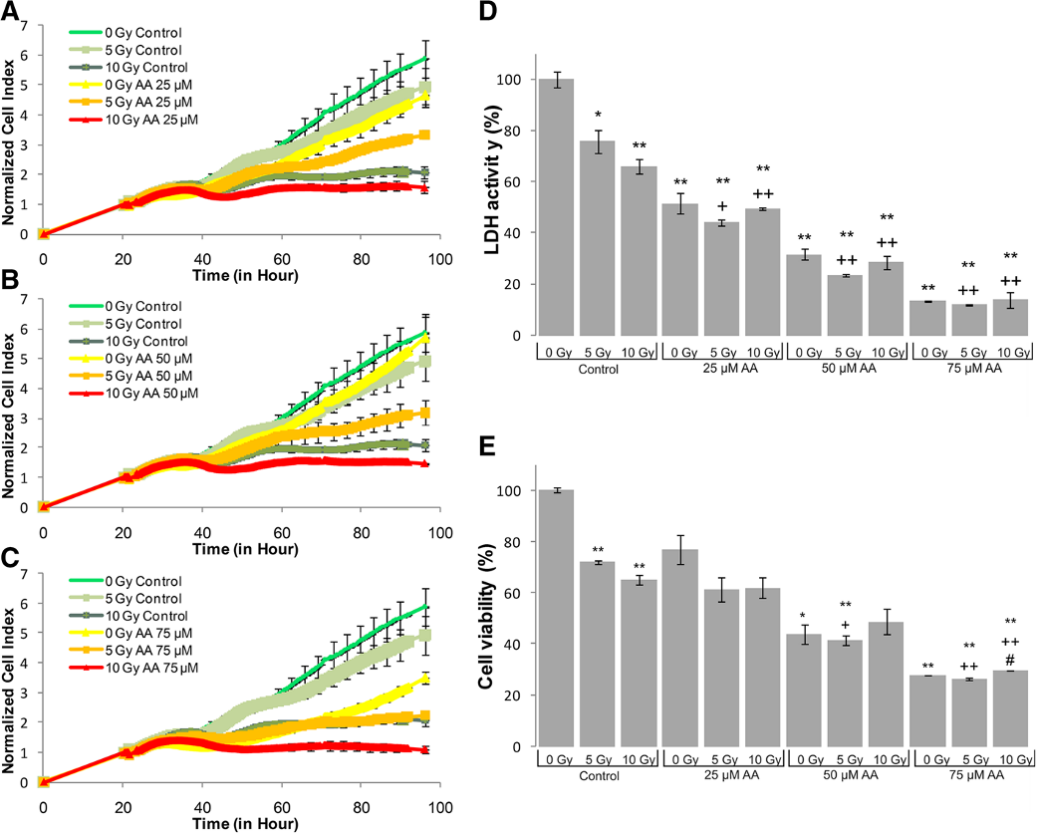
**Biochemical and biophysical assays on irradiated and AA treated glioma cell line.** The effect of AA on impedance based toxicity profiles **(A-C)**, LDH activity **(D)** and cell viability **(E)** on irradiated U87 MG cells. */** - significant (p < 0.05/ p < 0.01) alteration compared to control cells, +/++ - significant (p < 0.05/ p < 0.01) compared to cells exposed to irradiation (5 or 10 Gy, dose- matched), #/## - significant (p < 0.05/ p < 0.01) compared to cells subjected to AA (concentration matched).

**Figure 2:**
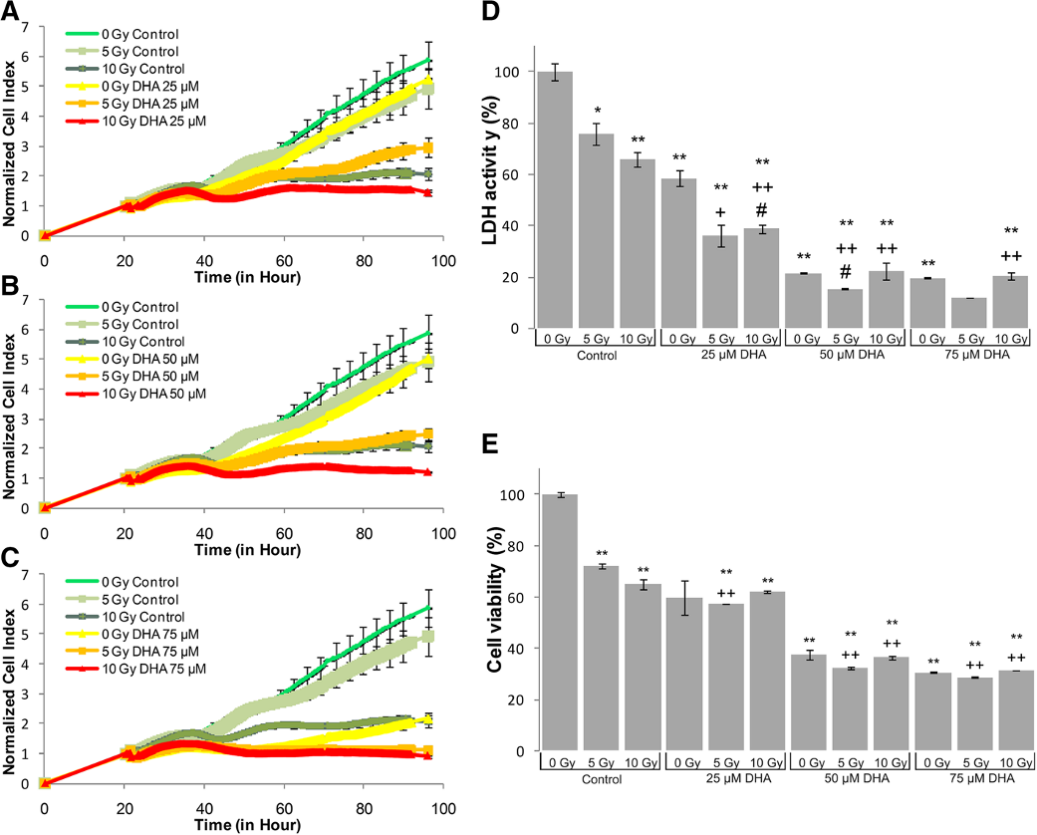
**Biochemical and biophysical assays on irradiated and DHA treated glioma cell line.** The effect of DHA on impedance based toxicity profiles **(A-C)**, LDH activity **(D)** and cell viability **(E)** on irradiated U87 MG cells.*/** - significant (p < 0.05/ p < 0.01) alteration compared to control cells, +/++ - significant (p < 0.05/ p < 0.01) compared to cells exposed to irradiation (5 or 10 Gy, dose- matched), #/## - significant (p < 0.05/ p < 0.01) compared to cells treated with DHA (concentration matched).

**Figure 3:**
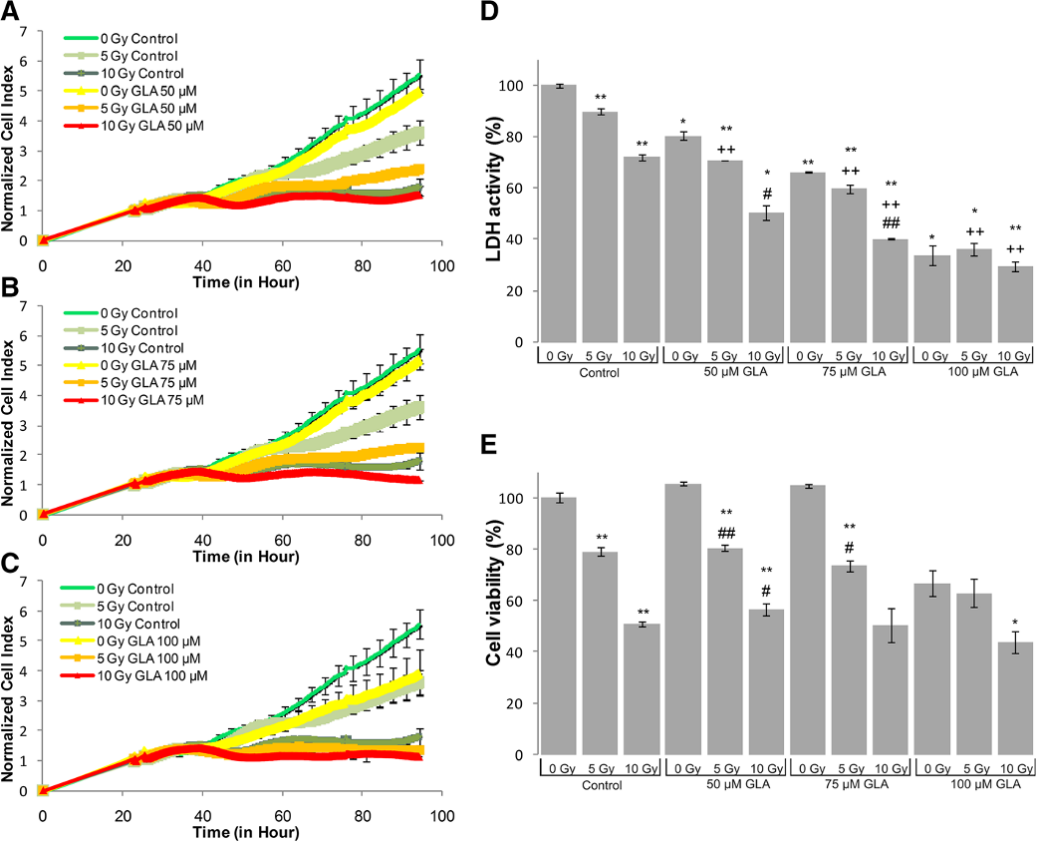
**Biochemical and biophysical assays on irradiated and GLA treated glioma cell line.** The effect of GLA on impedance based toxicity profiles **(A-C)**, LDH activity **(D)** and cell viability **(E)** on irradiated U87 MG cells. */** - significant (p < 0.05/ p < 0.01) alteration compared to control cells, +/++ - significant (p < 0.05/ p < 0.01) compared to cells exposed to irradiation (5 or 10 Gy, dose- matched), #/## - significant (p < 0.05/ p < 0.01) compared to cells treated with GLA (concentration matched).

**Figure 4:**
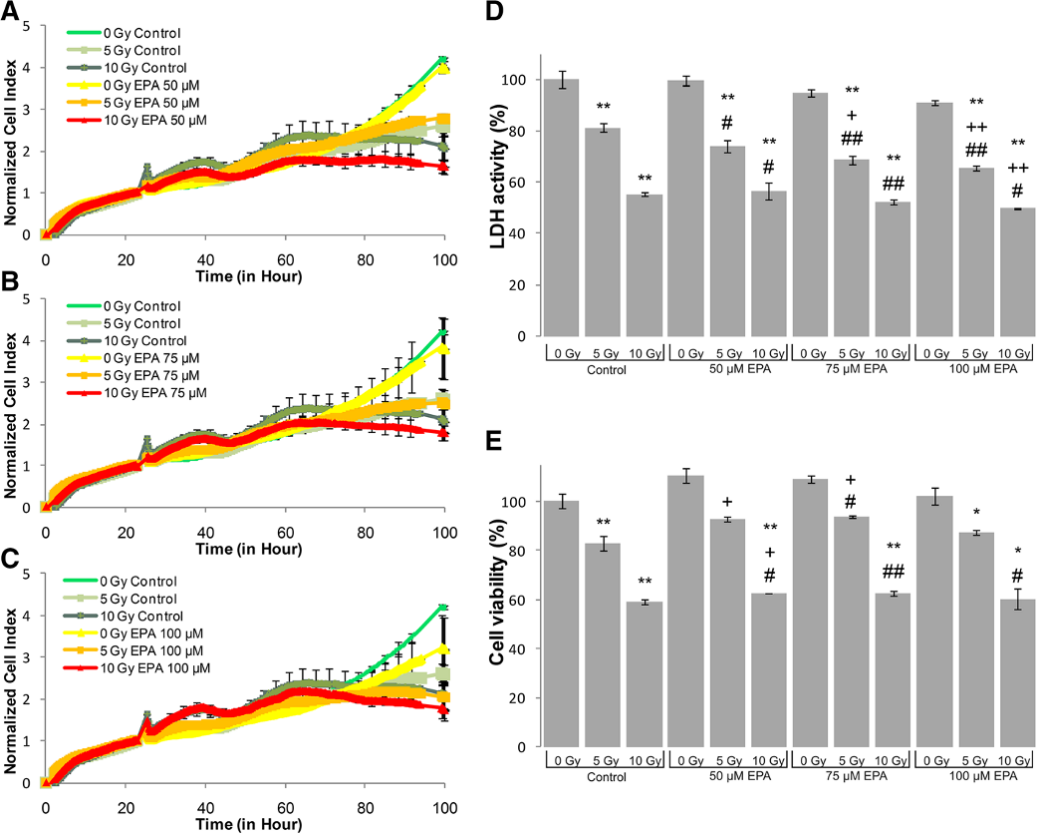
**Biochemical and biophysical assays on irradiated and EPA treated glioma cell line.** The effect of EPA on impedance based toxicity profiles **(A-C)**, LDH activity **(D)** and cell viability **(E)** on irradiated U87 MG cells. */** - significant (p < 0.05/ p < 0.01) alteration compared to control cells, +/++ - significant (p < 0.05/ p < 0.01) compared to cells exposed to irradiation (5 or 10 Gy, dose- matched), #/## - significant (p < 0.05/ p < 0.01) compared to cells treated with EPA (concentration matched).

**Figure 5:**
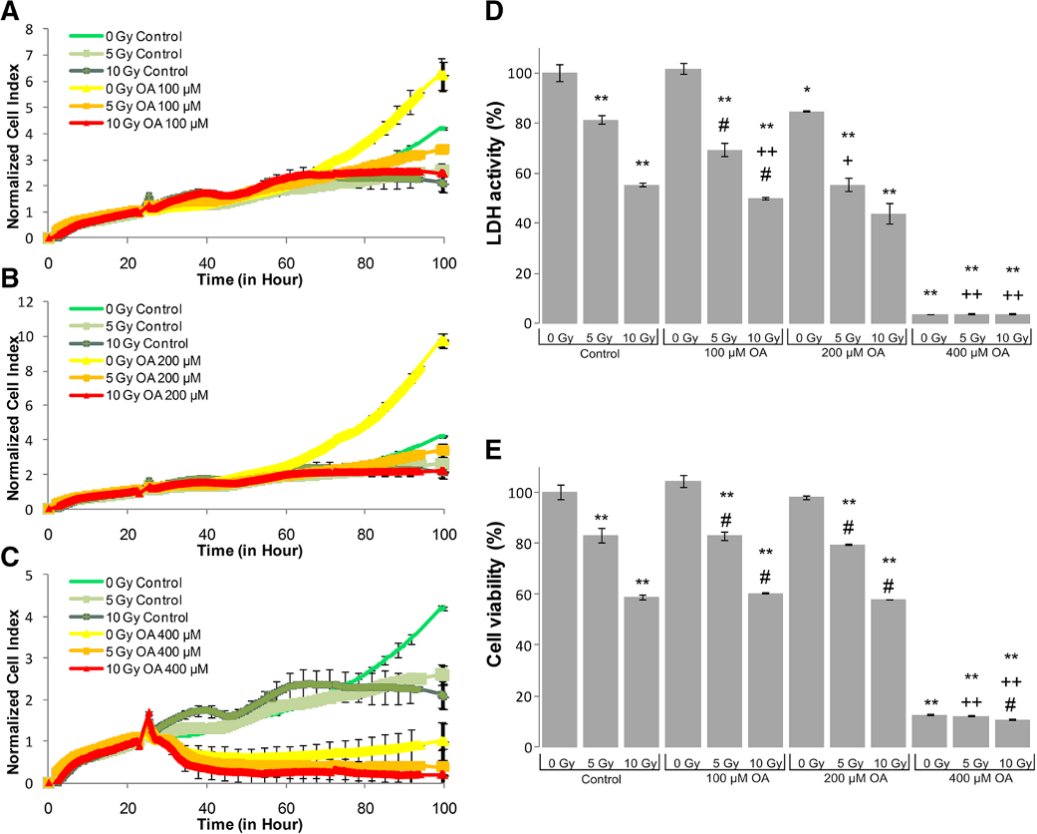
**Biochemical and biophysical assays on irradiated and OA treated glioma cell line.** The effect of OA on impedance based toxicity profiles **(A-C)**, LDH activity **(D)** and cell viability **(E)** on irradiated U87 MG cells.*/** - significant (p < 0.05/ p < 0.01) alteration compared to control cells, +/++ - significant (p < 0.05/ p < 0.01) compared to cells exposed to irradiation (5 or 10 Gy, dose- matched), #/## - significant (p < 0.05/ p < 0.01) compared to cells treated with OA (concentration matched).

LDH-, MTS assay and impedance based toxicity analysis (RT-CES assay) were performed on U87 MG cells to record the effects of UFA treatment, irradiation and their combination (Figures 1, 2, 3, 4 and 5).

The RT-CES assay permitted us to investigate the kinetics of cell growth and proliferation and determine the onset of changes on the cells treated and/or irradiated with UFAs. RT-CES results were validated with two biochemical end-point assays, LDH and MTS measurements. Those combined treatments that showed at least an additive effect compared to UFA treatment or irradiation alone were selected for further validation and analysis. For the evaluation of morphological changes and alterations in gene and miRNA expression induced by UFAs and/or irradiation a 48 hour incubation period was selected (Figures 1, 2, 3, 4 and 5).

### Analysis of cell metabolism and cell growth by real time and end point assays

The RT-CES assay permits non-invasive, dynamic monitoring of living cells [23, 25]. This is a real-time micro-electronic sensor-based platform, which measures the relative change of impedance of electrodes in individual wells of special culture plates. Attached cells in the wells change the impedance of the electrodes by acting as insulators, thus the assay enables the continuous measurement of cell attachment and proliferation [23, 25]. The effects of five selected fatty acids alone and in combination with two doses of irradiation (5 Gy and 10 Gy) are shown in Figures 1, 2, 3, 4 and 5. Fatty acid pre-treatment and irradiation were carried out 24 hours after cell seeding.

According to our RT-CES, LDH and MTS assay results, irradiation with 5 or 10 Gy changed cell proliferation, viability and LDH activity in a dose dependent manner (Figures 1, 2, 3, 4 and 5, green curves on panel A, and the first 3 bars on panel D and E).

Four of the tested five fatty acids, namely AA, DHA, GLA and OA decreased cell viability, while EPA had no effect in the tested concentration range.

LDH and MTS assays showed a well-defined concentration response when only AA treatment was applied (Figure 1). With the real time assay considerable change could only be recorded at 75 μM. All three assays showed an additive effect of the combined treatments at 5 Gy irradiation and 25 μM AA.

DHA treatment influenced cell index values considerably only at 75 μM, while an appreciable drop in cell viability and LDH activity was recorded at 25 μM concentration (Figure 2). Significant additive effects could not be detected in the MTS assay, while the 25 μM treatment combined with 5 or 10 Gy dose showed synergism in both the LDH and RT-CES assay.50 and 75 μM GLA did not alter the kinetics of cell index, while combination with 5 Gy irradiation reduced the rate of proliferation of U87 MG cells (Figure 3). GLA treatment and irradiation also influenced the MTS and LDH activity of U87 MG cells significantly (Figure 3). In case of exposure to 75 μM GLA and 10 Gy we noticed a strong additive effect on LDH activity.

Combined treatment of U87 MG cells with EPA and irradiation did not affect the impedance based toxicity profiles compared to irradiation alone (Figure 4). The LDH assay showed a significant decrease in signal at 75 and 100 μM EPA concentrations compared to 5 Gy, but this change was not confirmed by the other two assays.

In contrast with other UFAs 100-200 μM OA increased cell index values compared to respective controls, that reflect increased proliferation (Figure 5), however this was not confirmed by the end-point assays. The observed signal increase may be due to a change in cell morphology following OA administration. Treatment with 400 μM resulted in complete cell death confirmed by all three methods.

Based on the results presented above, we have chosen three PUFAs, namely, AA, DHA and GLA for further investigation.

### Morphological analysis of glioma cell line treated with AA, DHA, GLA and irradiation

**Figure 6:**
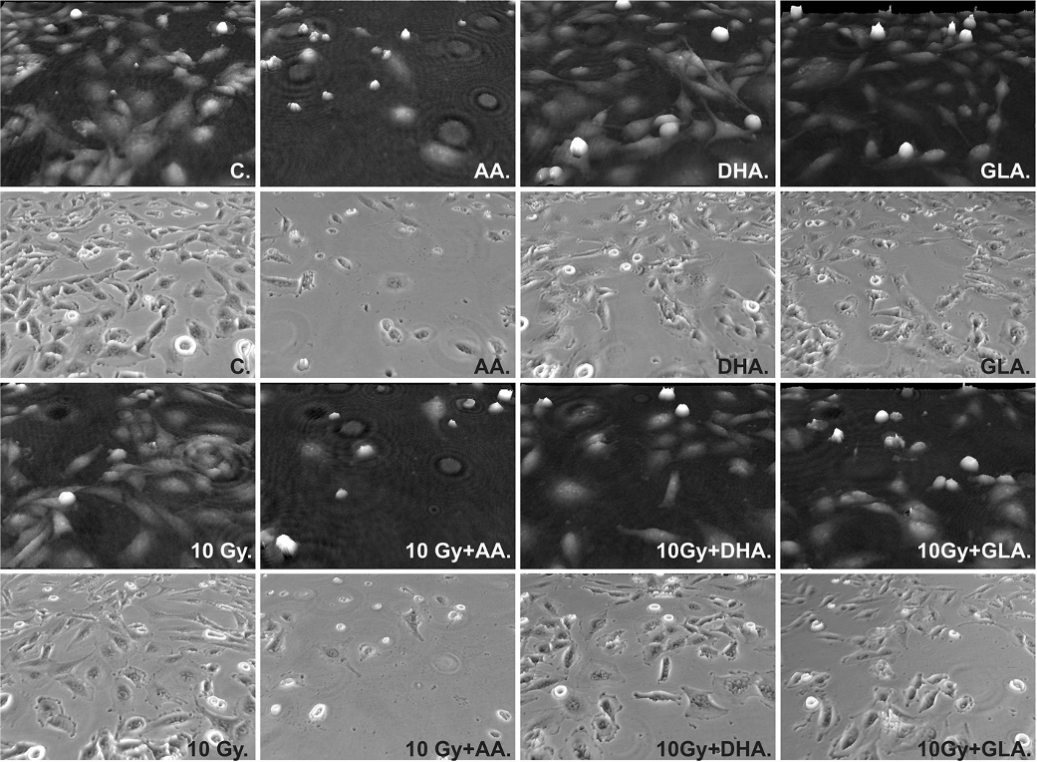
**Representative images of morphology of U87 MG cells treated with PUFAs and subjected to irradiation.** Phase contrast images were taken 48 hours after treatment with 25 μM AA, 25 μM DHA, 50 μM GLA and irradiation with 10 Gy.

Holographic and phase contrast (3-3 frames) pictures were taken with HoloMonitor™ M3 (Figure 6). 600,000 cells were exposed to 25 μM AA, 25 μM DHA or 50 μM GLA alone or in combination with irradiation (10 Gy).

**Figure 7:**
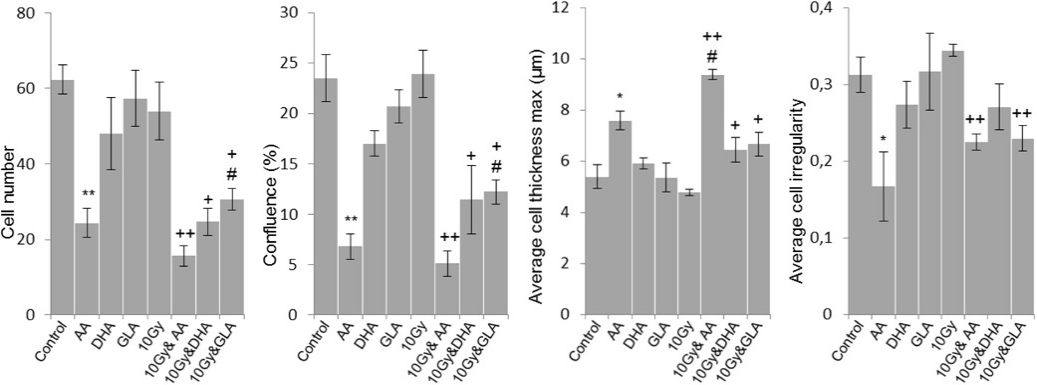
**The effect of PUFAs and irradiation on cell number, confluence, average cell thickness and average cell irregularity.** Cells were treated for 48 hours with 25 μM AA; 25 μM DHA and 50 μM GLA and a part of the samples were submitted to 10 Gy irradiation. Abbreviations: */** - significant (p < 0.05/p < 0.01) difference between control cells and treated cells, +/++ significant (p < 0.05/ p < 0.01) difference between cells exposed only to 10 Gy and U87 MG cells co-treated with PUFA and 10 Gy, #/## significant (p < 0.05/ p < 0.01) difference between cells treated solely with PUFAs and U87 MG cells co-treated with PUFA and 10 Gy (type and concentration matched).

Based on holographic and phase contrast images, treatment with 25 μM AA or the combination of 25 μM AA and 10 Gy was the most effective in terms of anti-proliferative effects, although irradiation did not cause a significant decrease in cell number and confluence compared to only AA treatment (Figures 6 and 7). DHA and GLA treatment enhanced the effect of irradiation, while alone they did not alter cell number and confluence (Figure 7). After a more thorough investigation of these parameters the synergistic effect of PUFA and irradiation is evident, exposure to 10 Gy did not alter cell number and confluence, while co-treatment induced a significant decrease in cell number (Figure 7). Two parameters (cell thickness and irregularity) indicating cell death also showed added effects of the combined treatment (Figure 7). Exposure to PUFAs and irradiation increased cell thickness while irregularity decreased. The alteration in the latter two parameters suggested that cells became rounded and started to detach from the surface showing a typical phenotype of dying cells. Exposure to AA, 10 Gy, the combination of AA + 10 Gy, DHA + 10 Gy and GLA + 10 Gy significantly increased cell thickness (Figure 7). Average cell irregularity is also diminished by AA alone, AA + 10 Gy, and GLA + 10 Gy treatment (Figure 7).

Our morphological analysis demonstrates that co-exposure to PUFAs and irradiation can cause synergistic changes in phenotypic parameters of U87 MG cells.

### Gene expression analysis of PUFA treated and irradiated U87 MG cells

In our previous paper we have shown that in several glioma cell lines (U373, GBM2, GBM5) exposure to AA, DHA and GLA differentially modify the expression of miRNAs and their corresponding target genes inducing apoptosis [7].

**Figure 8:**
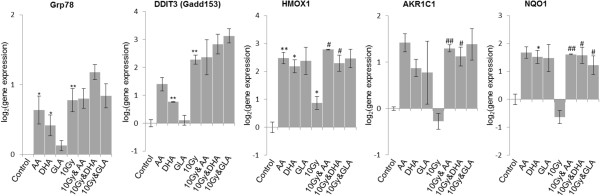
**Gene expression analysis of PUFA treated and irradiated glioma cell line.** U87 MG cells were irradiated with 10 Gy, treated with PUFAs, than incubated for 48 hours. Gene expression of oxidative stress related genes (*HMOX1*, *AKR1C1*, *NQO1*) and endoplasmic reticulum stress response genes (*GRP78*, *DDIT3*) were determined with RT-PCR. Abbreviations: AA - 25 μM arachidonic acid; DHA - 25 μM docosahexaenoic acid; GLA - 50 μM gamma linolenic acid. Abbreviations: */** significant (p < 0.05/ p < 0.01) difference between Ct values of control cells and treated cells; #/## - significant (p < 0.05/ p < 0.01) difference between Ct values of cells exposed to 10 Gy and U87 MG cells subjected to PUFAs and 10 Gy at the same time.

**Figure 9:**
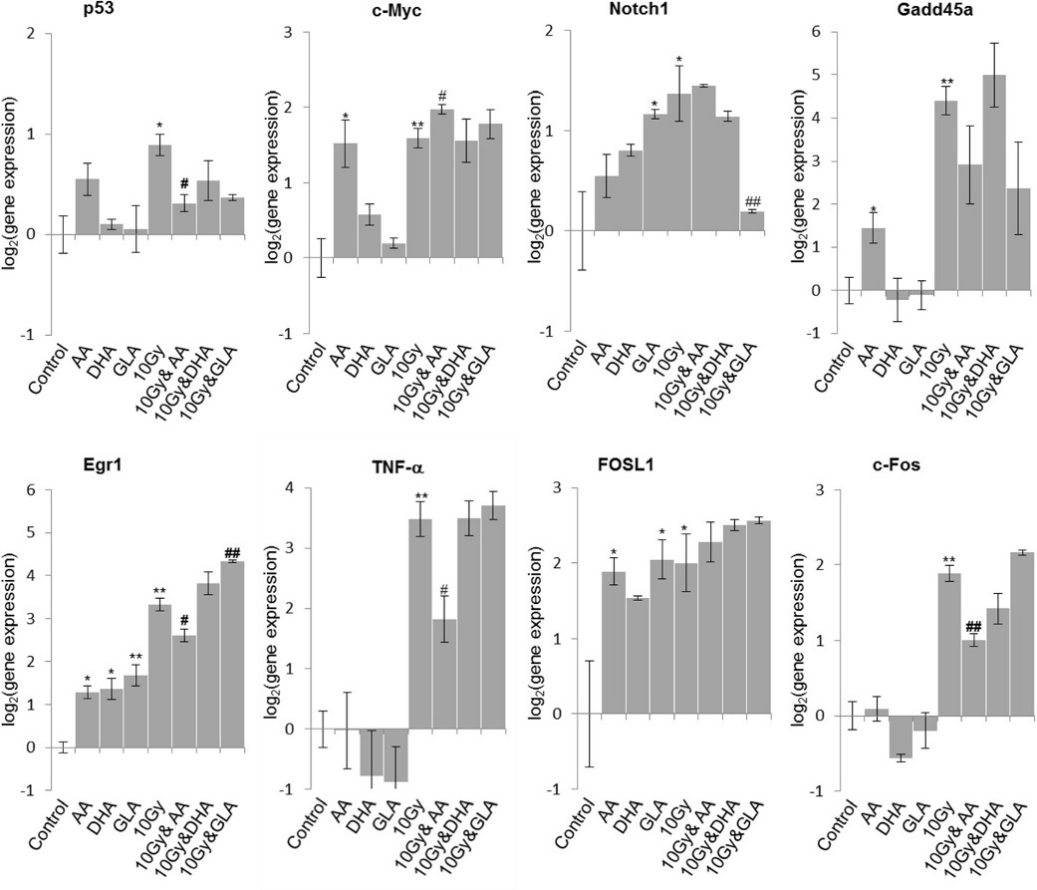
**Gene expression analysis of PUFA treated and irradiated glioma cell line.** U87 MG cells were irradiated with 10 Gy, treated with polyunsaturated fatty acids (PUFAs) and incubated for 48 hours. Gene expression of *c-MYC*, *TP53 (p53)*, potential therapeutic targets (*NOTCH1*, *GADD45A*, *EGR1*) and early response genes (*EGR1*,*TNF-α*, *FOSL1*, *c-FOS*) were determined with RT-PCR. Abbreviations: AA - 25 μM arachidonic acid; DHA - 25 μM docosahexaenoic acid; GLA - 50 μM gamma linolenic acid. Abbreviations: */** - significant (p < 0.05/ p < 0.01) difference between Ct values of control cells and treated cells. #/## - significant (p < 0.05/ p < 0.01) difference between Ct values of cells exposed to 10 Gy and U87 MG cells subjected to PUFAs and 10 Gy at the same time.

After treatment of U87 MG cells with PUFA or irradiation we investigated the expression of oxidative stress related genes (*HMOX1*, *AKR1C1*, *NQO1*), endoplasmic reticulum stress response genes (*GRP78*, *DDIT3*), early response genes (*EGR1*, *TNF-α*, *c-FOS*, *FOSL1*), an oncogene (*c-MYC)* and of *TP53, GADD45A* and *NOTCH1* (Figures 8 and 9). We noticed alteration in the expression of every gene at least in one condition: PUFA treatment or irradiation. PUFA treatment and 10 Gy had a significant effect in case of *EGR1*, *TNF-α*, *NOTCH1*, *c-FOS*, *c-MYC*, *TP53*, *HMOX1*, *AKR1C1* and *NQO1* compared to irradiated cells (Figures 8 and 9). Addition of PUFAs as adjuvants to 10 Gy did not alter the effect of irradiation on endoplasmic reticulum stress response (Figure 8, *GRP78* and *DDIT3*). On the other hand, the expression of oxidative stress response related genes (Figure 8, *HMOX1*, *AKR1C1* and *NQO1*) were significantly up-regulated due to co-exposure to PUFAs and 10 Gy compared to the case when cells were just irradiated.

Over-expression of *TP53* and *c-MYC* could be noticed when U87 MG cells were irradiated (Figure 9). When AA was added to 10 Gy, *TP53* expression decreased significantly, while *c-MYC* was up-regulated (Figure 9). In case of *c-MYC*, AA treatment was enough to significantly induce its expression.

Application of GLA or 10 Gy up-regulated *NOTCH1* expression. In contrast, after combined treatment *NOTCH1* expression was significantly lower (Figure 9). The expression of *GADD45A* increased significantly after exposure to AA or 10 Gy (Figure 9).

10 Gy caused significant over-expression in case of every early-response gene (*EGR1*, *TNF-α*, *FOSL1*, *c-FOS*) we investigated. This is likely due to their radiation sensitive CArG promoters (Figure 9). AA, DHA or GLA treatment increased *EGR1* expression significantly. When cells were co-treated with AA and 10 Gy, *EGR1*, *TNF-α*, *c-FOS* expression decreased significantly compared to the irradiated sample (Figure 9). This could not be noted in case of DHA and GLA. When cells were exposed to GLA and 10 Gy an additive effect was detected in *EGR1* expression (Figure 9). PUFA treatment did not influence *TNF-α* and *c-FOS* expression in a significant manner (Figure 9). PUFA treatment and application of PUFAs with irradiation induced over-expression of *FOSL1* (Figure 9).

We also investigated the effect of the selected PUFAs and irradiation on the following genes: *SIRT1*, *MMP14*, *TGFBI*, *TIMP3*, but alteration in the expression of these genes was not detected (data not shown).

Irradiation with 10 Gy and PUFA treatment did not affect the expression of miR-34a, miR-96, miR-148a, miR-148b and miR-152 significantly (Additional file 1: Figure S1). miR-146a was up-regulated when cells were treated with DHA, while its expression decreased when it was exposed to GLA (Additional file 1: Figure S1). In case of combined exposure to irradiation and GLA the expression increased significantly compared to irradiated or GLA treated cells. miR-181a expression significantly increased following DHA treatment (Additional file 1: Figure S1).

## Discussion

Radiotherapy is the most often applied treatment after surgical resection of glioblastoma. Application PUFAs as adjuncts enhance eradication of glioma cells. Numerous *in vitro* and *in vivo* experiments have shown that PUFAs may increase the tumoricidal effect of radiotherapy [19, 22, 26]. PUFAs have little or no cytotoxic effect on normal cells, and at the same time, they diminish the deteriorative effect of irradiation. In our study, we treated U87 MG cells with UFAs (AA, DHA, GLA, OA, EPA) at different concentrations and cell viability, LDH activity, cell growth, cell morphology and gene expression changes were analyzed. Besides classical end-point assays (LDH measurements and MTS assay) we used the RT-CES system for real-time cellular analysis. This label-free and non-invasive method measures impedance and determines cell index, an indicator of cell number, proliferation, viability, adherence and cell growth [23, 25].

We demonstrated that AA, DHA, GLA and OA treatment decreased the proliferation rate of U87 MG glioma cells and in correlation with the cytotoxic effects, decreased the total LDH activity that could be recorded (Figure 1, 2, 3 and 5). EPA is an exception: it did not decrease the proliferation rate and LDH activity at the tested concentration range (Figure 4).

AA treatment dramatically decreased cell viability and LDH activity after 72 hours (Figure 1). Based on similar effects against glioma cells AA was considered a possible therapeutic PUFA agent [27]. When cells are irradiated and treated with AA at the same time, LDH activity, mitochondrial dehydrogenase activity, were significantly decreased (Figure 1). We also detected a decrease in normalized cell index, which is an indicator of cell proliferation. This was more pronounced when AA was applied in combination with irradiation. From these results we assume that AA treatment would hold promise in glioblastoma radiotherapy as an adjunct.

Previously it was published that 20-50 μM DHA was cytotoxic to Neuro2a cells, and the concentration range below 10 μM inhibited apoptosis, without any detectable toxic effects [28]. We made similar observations: 25-75 μM DHA diminished the proliferation rate and altered the metabolism of U87 MG cells (Figure 2). DHA treatment had a distinct effect on medulloblastoma (DAOY and D283) cells compared to glioma cells (U87 MG and U138) regarding cell proliferation: it did not affect glioma cells, while it inhibited proliferation of medulloblastoma cells [29].

In concert with our results related to GLA (Figure 5), similar proliferation inhibition was reported with C6 glioma cells [30].They found that in tumors treated with GLA and EPA the mitochondrial membrane potential, an indicator of apoptosis, decreased significantly [30, 31]. In our study GLA diminished cell viability and LDH activity of U87 MG cells, and increased the radio sensitivity of this cell line. Similarly, GLA was found to be cytotoxic to rat 36B10 astrocytoma cells in other studies [32]. 10-50 μM GLA significantly increased cell proliferation at the outer layer of glioma spheroids, enhancing invasion [33]. In contrast, we found that 50-75 μM GLA did not alter the proliferation rate of U87 MG cells (Figure 3). It was reported that GLA selectively induced apoptosis in spheroids and concentrations that exceed 100 μM inhibited proliferation, thus reduced invasion [33]. Similarly, we found that 100 μM GLA diminished the proliferation rate of U87 MG cells. Interestingly, when 50-75 μM GLA was applied as adjunct to radiotherapy, proliferation and LDH activity of U87 MG cells were reduced (Figure 3). Previously, it was found that GLA acted selectively on tumor cells, it had low neurotoxicity and it may even protect normal tissue from the cytotoxic effect of irradiation or chemotherapy [10, 11]. Therefore, the additive effects of GLA with irradiation, its possible selectivity against tumor cells and even the protection of normal tissues against irradiation would make GLA an ideal candidate for combined therapy as previously indicated earlier [10, 11].

Interestingly, we found that at lower OA concentrations (100-200 μM) normalized cell index increased (Figure 5). This suggested elevated proliferation, although this was not confirmed with the end-point assays. At higher concentrations (400 μM) OA diminished cell proliferation soon after treatment as recorded by using the real-time cellular analysis (Figure 5). A similar concentration of OA (500 μM) influenced cell proliferation in a different manner depending on cell type: it inhibited cell growth on LNCaP prostate cells, it enhanced cell proliferation in case of breast cancer cell lines (MCF-7 and MDA-MB-231) and it had no effect on a non-tumorogenic epithelial cell line (MCF10A) [34, 35]. Our results showed no benefit using OA along with irradiation.

Previously, it was reported that EPA, similarly to GLA, protected rat hippocampus from the harmful effect of LPS-induced inflammation [36], therefore in case of additive effects of EPA and irradiation one could predict enhanced therapeutic effects. Under our conditions EPA treatment (50-100 μM) did not affect LDH activity and cell viability. Moreover, when it was used as an adjunct with 5 Gy or 10 Gy a significant, but very moderate change in cell metabolism could be detected (Figure 4). As assessed by using real-time cell analysis technology, EPA had no effect on normalized cell index of U87 MG cells even when it was applied in combination with 5 or 10 Gy irradiation (Figure 5). From these results we assume that EPA treatment would not be good candidate as an adjunct in glioblastoma radiotherapy.

Based on our observations on cell proliferation measurements and previously published data we could conclude that among the UFA we studied, DHA, GLA and AA may provide benefit as therapeutic adjuncts in the treatment of malignant brain tumor with radiation (results are summarized in Additional file 2: Table S2).

### Morphological analysis of glioma cell line treated with AA, DHA, GLA and irradiation

Holographic microscopy permits the label-free and non-invasive visualization of living cells. Furthermore, it allows the determination of cell number and confluence. An integrated image analysis algorithm makes it possible to measure more than forty parameters of each cell in a holographic image (cell volume, cell thickness, cell shape convexity, cell perimeter length, cell optical length, etc.) which reflects cytotoxicity [37]. During apoptosis, cell membrane permeability increases and the optical density of cells decreases, this changes their texture and the contrast becomes lower (http://www.phiab.se/products/holomonitor).U87 MG glioma cells were exposed to 25 μM AA, 25 μM DHA or 50 μM GLA alone or in combination with irradiation (10 Gy) and holographic and phase contrast images were recorded to detect morphological alterations following treatment (Figure 6).

Our results showed that PUFAs as adjuncts to a dose of 10 Gy significantly diminished cell number, confluence, and average cell irregularity, while average cell thickness increased significantly (Figure 7). The latter described parameters indicate cell rounding and loss of adherence, which indicates that the treatment had a cytotoxic effect on U87 MG cells.

Our results concerning cell number, confluence, average cell thickness and average cell irregularity imply that combined treatment of glioma cells with AA, DHA or GLA and radiotherapy would have inhibitory effects on invasion and metastasis.

### Gene expression analysis of PUFA treated and irradiated U87 MG cells

Several molecular targets for glioma treatment are subjects of clinical trials and under development [5, 29, 38]. Due to the complexity of glioma pathogenesis the application of more than one molecular target could be a solution for proper therapy. The foundations of an effective therapy would be the better knowledge of the affected genes and miRNAs in glioma pathogenesis. Because PUFAs are supposed to be radio sensitizing agents in glioblastoma treatment, the mRNA and miRNA expression analysis presented here emphasize several potential molecular targets (our results are summarized in Additional file 2: Table S3).

We found that AA, significantly increased *c-MYC* expression, just like 10 Gy, and combined exposure of U87 MG cells had an increased effect (Figure 8). Determination of *c-MYC* expression may serve as a prognostic value in glioblastoma, its expression was increased in approximately 70% of the cases [39]. Alteration of *c-MYC* expression influenced apoptosis, cell cycle progression and carcinogenesis [40]. In Jurkat and Raji cells oleic acid and linolenic acid induced over-expression of *c-MYC* after 24 hours [41, 42]. On U87 MG cells we detected significant over-expression only in case of treatment with 25 μM AA (Figure 8). Although *c-MYC* is an oncogene, its overexpression is correlated with a higher survival probability (P < 0.0001) [39]. This result suggests that combined therapy of AA and irradiation may be beneficial for glioblastoma treatment (Figure 8).

According to previous findings DHA did not change the total levels of TP53, impaired DNA binding of TP53 was observed in endothelial cells [43]. Under our conditions 10 Gy significantly increased the expression of *TP53* on U87 MG cells, while GLA and DHA did not influence its expression (Figure 8). If AA was added as adjunct to radiotherapy the expression of *TP53* was significantly decreased (Figure 8). In our previous paper we investigated the effect of a three-four times higher concentration of AA, DHA and GLA applied for a shorter incubation period on glioma cell lines [7]. We found that they altered the expression of *TP53* in GBM5 and U373 glioma cell lines, but not in GBM2 cell line [7], similarly to U87 MG cells observed in the present study. Differences in *TP53* expression changes could be due to the different TP53 status, the variability of overall *TP53* expression and relative levels of isoforms as these differences in glioblastoma are well documented [44].

One explanation of the beneficial effect of PUFAs would be that they may increase the activity of antioxidant enzymes [32]. The excess of reactive oxygen species induce lipid peroxidation and hydroperoxide generation in glioma cells, which decrease their viability and their sensitivity to irradiation [6, 32, 45]. Therefore, we evaluated the expression of *HMOX1*, *AKR1C1* and *NQO1* genes which have a role in the defense mechanism against oxidative stress.

HMOX1 is a heat-shock protein; it degrades heme to biliverdin, CO and iron [46]. HMOX1 inhibits apoptosis and inflammation, diminishes oxidative stress, enhances the rate of proliferation and playes a role in resistance to irradiation or chemotherapy [46–49]. *HMOX1* is a potential therapeutic target, it is over-expressed and facilitates angiogenesis in glioma and may influence the outcome of the disease [47, 50]. Irradiation induced *HMOX1* expression on pancreatic cancer cells [47]. We observed the same effect when we irradiated U87 MG cells with 10 Gy (Figure 8). Exposure to AA or DHA or 10 Gy combined with AA or with DHA also increased its expression in a significant manner (Figure 8).

*AKR1C1* encodes a drug-metabolizing enzyme; the level of expression of this gene may influence the prognosis of different cancers [51]. Temozolomide treatment significantly increased the expression of *AKR1C1* in U373 and T98G glioblastoma cells [51]. We noticed the same effect when U87 MG cells were exposed to irradiation and AA or GLA treatment (Figure 8).

When AA, DHA or GLA was added as adjunct, *NQO1* expression increased significantly, and treatment with DHA by itself also raised *NQO1* expression. The exact function of *NQO1* in cancer genesis is not yet determined, but it is known that it activates the apoptotic protein TP53 and it is a priority target of glioblastoma chemotherapy [52, 53].

In our study combined treatment of 50 μM GLA and irradiation reduced significantly the over-expression of *NOTCH1* which could be recorded when cells were subjected only to GLA or they were only irradiated (Figure 8). The main setback in radiotherapy is the radioresistance of cancer stem cells, which may be attributed to the Notch signaling pathway [5, 29]. Altered Notch activity was detected in several types of tumors; it mediates self-renewal of glioblastoma and influences the response to radiotherapy [5, 29, 54].

Endoplasmic reticulum (ER) stress response may be an indicator of the efficiency of glioma treatment [55–57]. We evaluated both elements of the ER stress response: the prosurvival arm (unfolded protein response (UPR) pathway) which is responsible for the alleviation of ER stress, and the proapoptotic arm, which is activated in case of intensive stress, when the UPR pathway is overwhelmed. The UPR pathway is represented by *GRP78*, while *DDIT3* (*GADD153*) stands for the proapoptotic arm of the ER stress response [55–58]. Under our conditions significant over-expression of *GRP78* could be recorded when U87 MG cells were treated with AA or DHA alone, or when cells were irradiated. Similar up-regulation could be observed when cells were treated in combination with irradiation and AA, DHA or GLA (Figure 8).

*GRP78* silencing delays glioma cell growth and sensitizes human glioblastoma cell lines to chemotherapy [56, 58]. *GRP78* is a prognostic marker; overexpression of *GRP78* increases radioresistance of glioblastomas [58]. Combination of PUFA treatment with irradiation did not decrease the overexpression of *GRP78* or of *DDIT3* (Figure 8), thus it seems that PUFAs radio sensitize U87 MG cells through other pathways than the ER stress response.

We examined the expression of *EGR1*, *TNF-α*, *c-FOS* and *FOSL1* that were proven to be early-response genes and were up-regulated due to ionizing radiation: [59]. *c-FOS*, *EGR1* and *FOSL1* contains a region with a serum response element (SRE) as promoter, which is responsible for the sensitivity of these genes to ionizing radiation [59, 60]. Ionizing radiation induces reactive oxygen species and up-regulates *EGR1*, a zinc-finger protein with six CArG elements, which regulates the transcription of genes involved in differentiation and cell growth [59–61]. AA, DHA and GLA up-regulated *EGR1* and treatment with GLA enhanced the effect of irradiation (Figure 8). In contrast, co-exposure with AA and 10 Gy increased *EGR1* expression in a significantly lower manner than application of 10 Gy by itself (Figure 8).

TNF-α is a growth promoting cytokine, which determines the outcome of glioblastoma [62]. At low TNF-α concentration glioma cells have a higher survival rate, while overexpression of *TNF-α* induces neuronal cell death [62]. Irradiation alone, and combined with DHA and GLA increased *TNF-*α expression significantly (Figure 8). AA treatment of irradiated U87 MG cells significantly decreased the overexpression of *TNF-*α compared to cells that were only irradiated (Figure 8). Thus, it seems that AA diminishes the harmful effect caused by irradiation induced *TNF-*α over-expression. Thus, this ω-6 fatty acid may have therapeutic effect when it is combined with irradiation, reducing possible side-effects.

In our study treatment of U87 MG cells with 25 μM AA, 50 μM GLA; irradiation or exposure to 10 Gy and PUFAs significantly increased the expression of *FOSL1* compared to control cells (Figure 9). Overexpression of *FOSL1* may cause carcinogenesis, and is a typical characteristic of glioma [63]. *FOSL1* over-expression induced differentiation, inhibited proliferation, growth and reduced tumorogenicity of C6 glioma cell line, so it may be a potential target for glioma treatment [64].

*c-FOS* also contains serum response elements in its promoter [59, 60]. c-FOS, is an oncogenic transcription factor, which regulates PKC-mediated signaling pathways [59], and it can induce carcinogenesis [65]. Just as in case of *TNF-α*, when we treated U87 MG cells with 25 μM AA, it significantly decreased the overexpression of *c-FOS*, which is otherwise induced by irradiation (Figure 9).

*GADD45A* is a target for therapeutic interventions in cancer [66]. Exposure to 25 μM AA or 10 Gy significantly increased the expression of *GADD45A* (Figure 9). As a consequence of *GADD45A* overexpression TP53 is phosphorylated and it stabilizes TP53 after DNA damage [66].

While different tumor types present specific microRNA signatures, several microRNAs are deregulated in glioblastoma, suggesting their involvement in the basic processes of tumorigenesis and response to therapy [67]. To further analyze the mechanism of action of AA, DHA and GLA in combination with irradiation, miRNA expression levels were evaluated. Irradiation with 10 Gy and PUFA treatment did not alter significantly the expression of miR-34a, miR-96, miR-148a, miR-148b and miR-152. However, when cells were treated with DHA miR-146a was significantly up-regulated. Interestingly, its expression decreased when it was exposed to GLA. In case of combined exposure to irradiation and GLA the expression of miR-146a increased significantly compared with GLA alone or with irradiation. Recently it was shown that miR-146a suppresses gastric cancer cell invasion and metastasis *in vitro* and *in vivo* [68]. From our data it would be interesting to investigate the differential effects of DHA and GLA on miR-146a in relevance with metastatic potential of glioblastoma, especially that GLA was the only PUFA which, in combination with radiation, could induce its expression suggesting potent antimetastatic effects.

## Conclusions

Our biophysical, biochemical assays, 3D morphological and gene expression analysis confirmed that PUFA treatment enhances the radiosensitivity of glioma cells. Irradiation and PUFA treatment influenced significantly the expression of several potential therapeutic targets, *EGR1*, *HMOX1*, *NOTCH1*, *GADD45A* and *NQO1*, in a favorable manner. Based on our results, out of the five investigated UFAs (AA, DHA, GLA, EPA and OA) AA and GLA had the most significant additive cytotoxic effects with irradiation. However, for therapeutic applications further investigations are necessary.

## Material and methods

### Cell lines and culturing conditions

U87 MG (ATCC HTB-14^TM^) glioma cells were cultured at 37°C in 5% CO_2_ atmospheric pressure in DMEM supplemented with 10% FCS. Cells were plated in 16-well e-plates (Roche, Hungary), 96-well culture plates, T25 or T100 flasks at various densities depending on the type and set-up of the experiment. UFAs were administered 1 hour prior to irradiation. The added UFAs were present in the medium throughout the whole incubation interval, 24, 48, 72 or 100 hours, respectively. The UFAs could incorporate into the membranes or enter the cell until the end of the experiment. Cells were treated with the following UFAs: AA (Cayman Chemical Company, San Diego, California), EPA (Sigma-Aldrich, Budapest, Hungary), DHA (Cayman Chemical Company), GLA (Ubichem Research, Budapest, Hungary), OA (Sigma-Aldrich). Then cells were subjected to a dose of 5 or 10 Gy and incubated for 24, 48 or 72 hours.

### Irradiation

A Teragam K-01 cobalt unit was used (average energy 1.25MeV, SID = 80 cm) to irradiate cells dispensed in tissue culture plates and flasks. The plates or flasks were surrounded by water equivalent material at each side and placed between two PMMA slabs of 2 cm thickness to ensure the necessary build-up material. The isocenter was positioned in the geometrical centers of the plates. One half of the planned dose was delivered with a downward 20 × 20 cm beam (gantry angle 0 degrees), while the other half with an upward beam (gantry angle 180 degrees) to maximize the field homogeneity. The delivered doses were 0, 5 and 10 Gy respectively. Irradiation time correction factors due to the decay of the cobalt-60 source have been applied.

### Biochemical assays

#### LDH assay

U87 MG cells were seeded at 2000 cells/well density, incubated for 24 hours, treated with UFAs and one hour later, exposed to irradiation. 72 hours later media was removed from the wells and cells were washed with PBS. Following total cell lysis with 70 μL of 1% Triton X-100 (Sigma-Aldrich) in PBS, 70 μL LDH reagent was added (Roche). After 10 minutes, absorbance was measured at 490 nm.

#### MTS assay

Plating, treatment and incubation period was the same as in the case of the LDH assay. After 72 hour incubation, 20 μL of PMS:MTS (1:20) solution was added to the cells. 1 hour later, absorbance was measured at 490 nm.

#### xCELLigence assay

Impedance based real-time cell electronic sensing (RT-CES) assay was performed with xCELLigence RTCA instrument [24]. Cells were plated in 16-well e-plates at 2000 cells/well density. Next day cells were treated with UFAs, irradiated and monitored for 72 hours. Measurements were recorded every 10 minutes. Cell index values were normalized to the time point prior to UFA treatment.

### Holographic imaging

For holographic imaging 600,000 cells were plated in T25 flasks. 24 hours later the corresponding cells were exposed to a dose of 10 Gy and/or pre-treated with 25 μM AA, 25 μM DHA or 50 μM GLA and further incubated for 48 hours. Images were recorded with HoloMonitor™ M3 (Phase Holographic Imaging AB, Lund, Sweden). Phase contrast and holographic images were taken of three representative frames. The software of the apparatus counts cells and uses specific algorithms to define their outlines. It measures 43 parameters, for each cell, e.g.: confluence, cell area, cell optical path length, cell roughness, texture, volume, irregularity, etc.

All integral cells were taken into consideration in all frames. We applied paired Student’s t-test for significance analysis.

### Samples for gene and miRNA expression

600,000 U87 MG cells were seeded in T100 flasks and incubated for 24 hours. Cells were pre-treated with 25 μM AA, 25 μM DHA or 50 μM GLA and subjected to irradiation.

### Nucleic acid isolation

Columns, binding buffer and wash buffer were used from the Viral RNA extraction kit (Bioneer, Daejon, South Korea). Binding buffer 1 (2:1 etanol: binding buffer) and binding buffer 2 (5:1 etanol: binding buffer) was prepared. Cells were washed with PBS, incubated in lysis buffer (Accuzol™ Total RNA Extraction Solution, Bioneer, Daejon) for 5 minutes. The lysate was collected. The upper aqueous phase was collected after addition of dichloromethane. Binding buffer 1 was added to the sample, and then transferred through columns. The flow-through was collected for miRNA isolation as described previously [25]. The columns were treated with DNase (Omega bio-tek, Norcross, Georgia, USA). Binding buffer 2 was added to the flow-through, and the mixture was transferred through another binding column. After two subsequent washing steps, RNA and miRNA was eluted in RNase free-water.

The quality and quantity of the isolated miRNA and RNA was measured with NanoDrop1000 Version 3.8.1. (Thermo Fisher Scientific, Wilmington, USA).

### RNA expression

Reverse transcription from total RNA was performed with the High Capacity cDNA Reverse Transcription Kit as recommended by Applied Biosystems® (Life Technologies, Foster City, CA, USA). cDNA was diluted 18 times. Gene expression was measured with Platinum SYBR Green qPCR SuperMix (Invitrogen). 4.5 μL template cDNA was added to 5.5 μL Mastermix. QRT-PCR was performed on a RotorGene 3000 instrument (Corbett Life Science, QIAGEN) with gene-specific primers as previously described [69]. Primer sequences are presented in Additional file 3: Table S1. The final primer concentration was 250 nM. Ct values were determined with Rotor-Gene Version 6.0 (Corbett Life Science). The PCR protocol was the following: 1. 95°C 2 min; 2. 95°C 15 sec; 3. 60°C 45 sec; with 60 cycles. After cycling melting curves were recorded. Primer specificity was verified by comparing Tm values of the products and running non-template controls. Gene expression was normalized to HPRT1 and PPIA expression. p-values were determined with Welch’s ttest.

### miRNA expression

200 ng miRNA was transcribed with 5x TaqMan RT assay primer (Life Technologies) mix and high capacity cDNA Reverse Transcription Kit (Life Technologies) with the following protocol: 1. 16°C 30 min; 42°C 30 min; 85°C 5 min.

miRNA expression was determined with FastStart TaqMan Probe Master (Roche) and with TaqMan primers (20x, Life Technologies) with Exicycler 96 Real-Time Quantitative Thermal Block (Bioneer). Reagent composition for one sample is the following: 5 μL FastStart TaqMan Probe Master; 3.5 μL water; 0.5 μL primer and 1 μL cDNA with the following PCR protocol: heat-start at 95°C for 15 min; 50 cycles of 95°C for 15 sec; 60°C for 1 min.

Ct values of miRNA expression were normalized for the median of all miRNA measured on the same sample. Significance was determined with Welch’s t-test.

## Electronic supplementary material

Additional file 1: Figure S1: miRNA expression analysis of PUFA treated and irradiated glioma cell line. U87 MG cells were irradiated with 10 Gy, treated with polyunsaturated fatty acid (PUFAs) and incubated for 48 hours. miRNA expression was measured with RT-PCR. Abbreviations: AA- 25 μM arachidonic acid; DHA - 25 μM docosahexaenoic acid; GLA - 50 μM gamma linolenic acid. Abbreviations: */** - significant (p < 0.05/ p < 0.01) difference between Ct values of control cells and treated cells. #/## - significant (p < 0.05/ p < 0.01) difference between cells exposed only to 10 Gy and U87 MG cells treated with PUFA and 10 Gy. (TIFF 3 MB)

Additional file 2: Table S2: Summary of the effect of UFA treatment on U87 MG glioma cell line detected by RT-CES, LDH and MTS assay. **Table S3.** Summary of changes in cell morphology, in mRNA and in miRNA expression due to PUFA treatment and/or irradiation. (DOCX 21 KB)

Additional file 3: Table S1: List and sequence of primers used for gene expression analysis. (DOCX 17 KB)

## Abbreviations

AA: Arachidonic acid
DHA: Docosahexaenoic acid
miRNA: Micro RNA
MTS: (3-(4,5-dimethylthiazol-2-yl)-5-(3-carboxymethoxy phenyl)-2-(4-sulfophenyl)-2H-tetrazolium
EPA: Eicosapentaenoic acid
GLA: Gamma linolenic acid
LDH: Lactate dehydrogenase
OA: Oleic acid
PUFA: Polyunsaturated fatty acids
UFA: Unsaturated fatty acids
