# Supplementary figures and images for: Combination of unsaturated fatty acids and ionizing radiation on human glioma cells: cellular, biochemical and gene expression analysis
Source: Lipids Health Dis. 2014 Sep 2;13:142. doi: 10.1186/1476-511X-13-142 (PMC4176829; doi:10.1186/1476-511X-13-142)

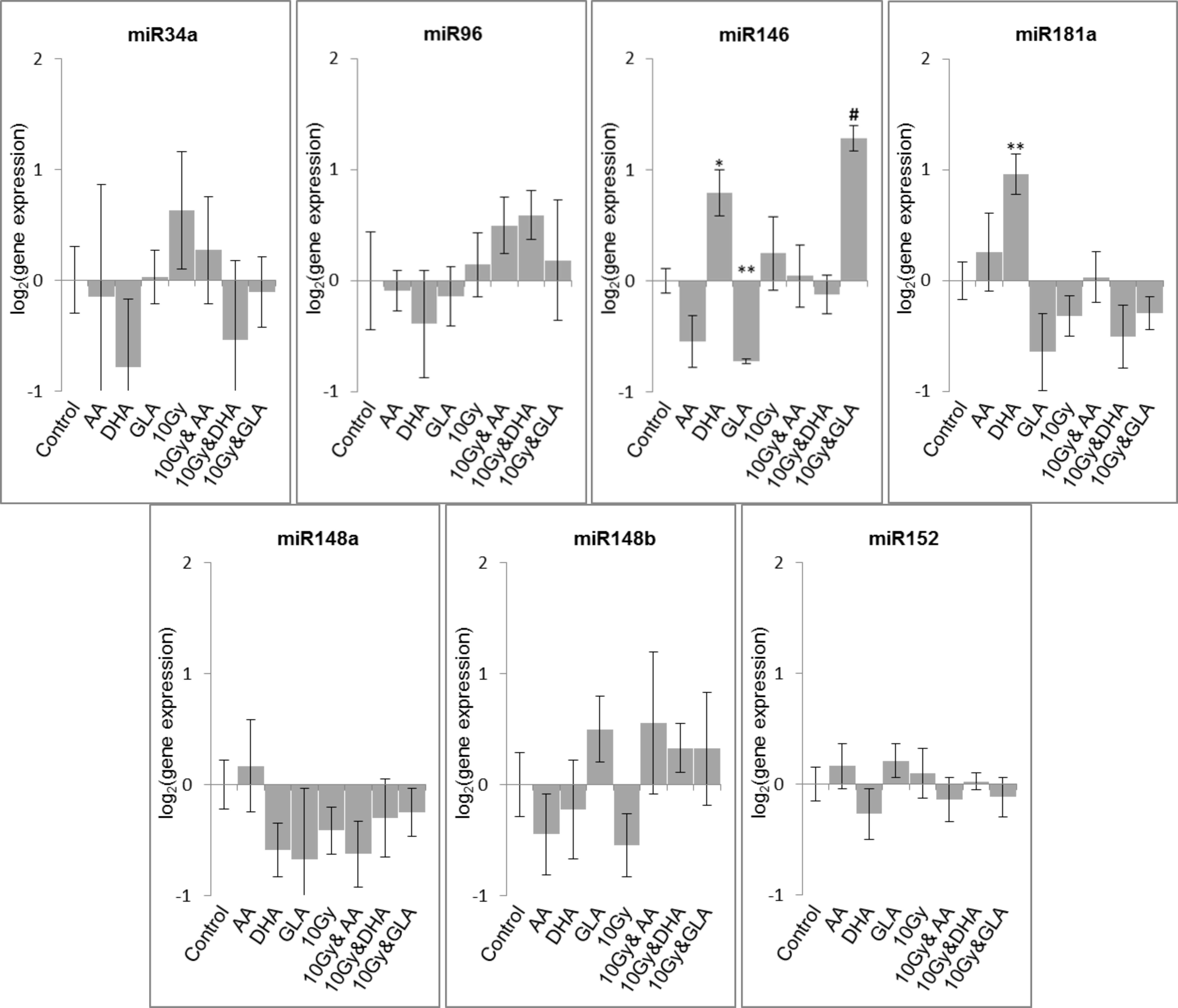

Supplement: Supplementary file 1 — Additional file 1: Figure S1: miRNA expression analysis of PUFA treated and irradiated glioma cell line. U87 MG cells were irradiated with 10 Gy, treated with polyunsaturated fatty acid (PUFAs) and incubated for 48 hours. miRNA expression was measured with RT-PCR. Abbreviations: AA- 25 μM arachidonic acid; DHA - 25 μM docosahexaenoic acid; GLA - 50 μM gamma linolenic acid. Abbreviations: */** - significant (p < 0.05/ p < 0.01) difference between Ct values of control cells and treated cells. #/## - significant (p < 0.05/ p < 0.01) difference between cells exposed only to 10 Gy and U87 MG cells treated with PUFA and 10 Gy. (TIFF 3 MB) [file 12944_2014_1130_MOESM1_ESM.tiff]
