# Supplementary material for: Combination of unsaturated fatty acids and ionizing radiation on human glioma cells: cellular, biochemical and gene expression analysis
Source: Lipids Health Dis. 2014 Sep 2;13:142. doi: 10.1186/1476-511X-13-142 (PMC4176829; doi:10.1186/1476-511X-13-142)
Supplement: Supplementary file 2 — Additional file 2: Table S2: Summary of the effect of UFA treatment on U87 MG glioma cell line detected by RT-CES, LDH and MTS assay. Table S3. Summary of changes in cell morphology, in mRNA and in miRNA expression due to PUFA treatment and/or irradiation. (DOCX 21 KB) [file 12944_2014_1130_MOESM2_ESM.docx]

Additional file 2: **Table S2: Summary of the effect of UFA treatment on U87 MG glioma cell line detected by RT-CES, LDH and MTS assay.** ↑ - proliferation/ LDH activity/ cell viability increased compared to control cells (0 μM UFA; 0 Gy); ↓ - proliferation/ LDH activity/ cell viability increased compared to control cells (0 μM UFA; 0 Gy); ~ - no significant difference could be detected between control cells (0 μM UFA; 0 Gy) and the respective treatment. * - synergism could be detected between UFA treatment and radiation. LDH activity and cell viability are expressed in percentage compared to control cells.

| **UFA type, concentration, and dose of irradiation** | **RT-CES** | **LDH (%)** | **MTS (%)** |
| --- | --- | --- | --- |
| 0 Gy 25 μM AA | ~ | 51.53 | 76.90 |
| 5 Gy 25 μM AA | ↓ | 44.05 | 61.42 |
| 10 Gy 25 μM AA | ↓ | 49.36 | 61.84 |
| 0 Gy 50 μM AA | ~ | 31.65 | 43.87 |
| 5 Gy 50 μM AA | ↓ | 23.41 | 41.44 |
| 10 Gy 50 μM AA | ↓ | 28.43 | 48.67 |
| 0 Gy 75 μM AA | ↓ | 13.3 | 27.48 |
| 5 Gy 75 μM AA | ↓ | 11.86 | 26.22 |
| 10 Gy 75 μM AA | ↓ | 13.74 | 29.49 * |
| 0 Gy 25 μM DHA | ~ | 58.49 | 59.83 |
| 5 Gy 25 μM DHA | ↓ | 36.27 | 57.29 |
| 10 Gy 25 μM DHA | ↓ | 38.85 * | 62.05 |
| 0 Gy 50 μM DHA | ~ | 21.5 | 37.58 |
| 5 Gy 50 μM DHA | ↓ | 15.52 * | 32.29 |
| 10 Gy 50 μM DHA | ↓ | 22.4 | 36.36 |
| 0 Gy 75 μM DHA | ↓ | 19.64 | 30.6 |
| 5 Gy 75 μM DHA | ↓ | 11.75 | 28.75 |
| 10 Gy 75 μM DHA | ↓ | 20.44 | 31.24 |
| 0 Gy 50 μM GLA | ~ | 80.31 | 105.41 |
| 5 Gy 50 μM GLA | ↓ | 70.72 | 80.31 |
| 10 Gy 50 μM GLA | ↓ | 50.33 | 56.65 |
| 0 Gy 75 μM GLA | ~ | 66.14 | 104.64 |
| 5 Gy 75 μM GLA | ↓ | 59.66 | 73.51 |
| 10 Gy 75 μM GLA | ↓ | 40.13 * | 50.61 |
| 0 Gy 100 μM GLA | ↓ | 33.68 | 66.61 |
| 5 Gy 100 μM GLA | ↓ | 36.29 | 62.78 |
| 10 Gy 100 μM GLA | ↓ | 29.6 | 43.72 |
| 0 Gy 50 μM EPA | ~ | 99.67 | 110.56 |
| 5 Gy 50 μM EPA | ~ | 74.06 | 92.78 |
| 10 Gy 50 μM EPA | ~ | 56.41 | 62.8 |
| 0 Gy 75 μM EPA | ~ | 95 | 109.06 |
| 5 Gy 75 μM EPA | ~ | 68.67* | 93.7 |
| 10 Gy 75 μM EPA | ~ | 52.33 | 62.57 |
| 0 Gy 100 μM EPA | ~ | 91.22 | 102.16 |
| 5 Gy 100 μM EPA | ~ | 65.71* | 87.31 |
| 10 Gy 100 μM EPA | ~ | 49.7* | 60.32 |
| 0 Gy 100 μM OA | ↑ | 101.71 | 104.34 |
| 5 Gy 100 μM OA | ~ | 69.36 | 82.88 |
| 10 Gy 100 μM OA | ~ | 50.07 * | 60.44 |
| 0 Gy 200 μM OA | ↑ | 84.88 | 98.07 |
| 5 Gy 200 μM OA | ~ | 55.46 | 79.49 |
| 10 Gy 200 μM OA | ~ | 43.85 | 57.79 |
| 0 Gy 400 μM OA | ↓ | 3.52 | 12.68 |
| 5 Gy 400 μM OA | ↓ | 3.85 | 12.28 |
| 10 Gy 400 μM OA | ↓ | 3.81 | 10.66* |

Additional file 2: **Table S3: Summary of changes in cell morphology, in mRNA and in miRNA expression due to PUFA treatment and/or irradiation.** Cells were treated for 48 hours with 25 μM AA; 25 μM DHA and 50 μM GLA and a part of the samples were irradiated with a dose of 10 Gy.

**Abbreviations:** the given parameter (cell number/ confluence/ average cell thickness/ average cell irregularity) or expression of gene/miRNA changed in the direction indicated by the following symbols: ↑ - increased; ↓ - decreased; ~ - did not change significantly. Samples exposed solely to PUFA or 10 Gy are compared to control (0 Gy, 0 μM PUFA); while those that are treated with both are compared to irradiated ones (10 Gy, 0 μM PUFA).

|  | Cell number | Conflu-ence | Average cell thickness | Average cell irregular-rity | Grp78 | DDIT3 | HMOX1 | AKR1C1 |
| --- | --- | --- | --- | --- | --- | --- | --- | --- |
| AA | ↓ | ↓ | ↑ | ↓ | ↑ | ~ | ↑ | ~ |
| DHA | ~ | ~ | ~ | ~ | ↑ | ↑ | ↑ | ~ |
| GLA | ~ | ~ | ~ | ~ | ~ | ~ | ~ | ~ |
| 10Gy | ~ | ~ | ~ | ~ | ↑ | ↑ | ↑ | ~ |
| 10Gy+AA | ↓ | ↓ | ↑ | ↓ | ~ | ~ | ↑ | ↑ |
| 10Gy+DHA | ↓ | ↓ | ↑ | ~ | ~ | ~ | ↑ | ↑ |
| 10Gy+GLA | ↓ | ↓ | ↑ | ↓ | ~ | ~ | ~ | ~ |

|  | NQO1 | TP53 | c-Myc | Notch1 | Gadd45a | Egr1 | TNF-α | FOSL1 |
| --- | --- | --- | --- | --- | --- | --- | --- | --- |
| AA | ~ | ~ | ↑ | ~ | ↑ | ↑ | ~ | ↑ |
| DHA | ↑ | ~ | ~ | ~ | ~ | ↑ | ~ | ~ |
| GLA | ~ | ~ | ~ | ↑ | ~ | ↑ | ~ | ↑ |
| 10Gy | ~ | ↑ | ↑ | ↑ | ↑ | ↑ | ↑ | ↑ |
| 10Gy+AA | ↑ | ↓ | ↑ | ~ | ~ | ↓ | ↓ | ~ |
| 10Gy+DHA | ↑ | ~ | ~ | ~ | ~ | ~ | ~ | ~ |
| 10Gy+GLA | ↑ | ~ | ~ | ↓ | ~ | ↑ | ~ | ~ |

|  | c-Fos | miR34a | miR96 | miR146 | miR181a | miR148a | miR148b | miR152 |
| --- | --- | --- | --- | --- | --- | --- | --- | --- |
| AA | ~ | ~ | ~ | ~ | ~ | ~ | ~ | ~ |
| DHA | ~ | ~ | ~ | ↑ | ↑ | ~ | ~ | ~ |
| GLA | ~ | ~ | ~ | ↓ | ~ | ~ | ~ | ~ |
| 10Gy | ↑ | ~ | ~ | ~ | ~ | ~ | ~ | ~ |
| 10Gy+AA | ↓ | ~ | ~ | ~ | ~ | ~ | ~ | ~ |
| 10Gy+DHA | ~ | ~ | ~ | ~ | ~ | ~ | ~ | ~ |
| 10Gy+GLA | ~ | ~ | ~ | ↑ | ~ | ~ | ~ | ~ |
