# Supplementary material for: Combination of unsaturated fatty acids and ionizing radiation on human glioma cells: cellular, biochemical and gene expression analysis
Source: Lipids Health Dis. 2014 Sep 2;13:142. doi: 10.1186/1476-511X-13-142 (PMC4176829; doi:10.1186/1476-511X-13-142)
Supplement: Supplementary file 3 — Additional file 3: Table S1: List and sequence of primers used for gene expression analysis. (DOCX 17 KB) [file 12944_2014_1130_MOESM3_ESM.docx]

**Additional file 3: Table S1: List and sequence of primers used for gene expression analysis.**

| *c-FOS* | Fw | AGGACCTTATCTGTGCGTGAAAC |
| --- | --- | --- |
|  | Rv | CCACACATGGATGCTTTCAAGT |
| *c-MYC* | Fw | CTGGATCGGGGTAAAGTGAC |
|  | Rv | AAAAACCATTCCCGTTTTCC |
| *DDIT3* | Fw | CAGAGCTGGAACCTGAGGAG |
|  | Rv | TGGATCAGTCTGGAAAAGCA |
| *EGR1* | Fw | AGCCCTACGAGCACCTGAC |
|  | Rv | GGTTTGGCTGGGGTAACTG |
| *FOSL1* | Fw | AACCGGAGGAAGGAACTGAC |
|  | Rv | CTGCAGCCCAGATTTCTCA |
| *GADD45A* | Fw | TTTGCAATATGACTTTGGAGGA |
|  | Rv | CATCCCCCACCTTATCCAT |
| *GRP78* | Fw | AGCAGGGCCTTCACCAAT |
|  | Rv | CGTCGCCTACTCGGCTTAT |
| *MMP14* | Fw | TCCAAGGAAGGAGCCTGAG |
|  | Rv | CCCATCCAAGGCTAACATTC |
| *NOTCH1* | Fw | ACGCACAAGGTGTCTTCCA |
|  | Rv | AGGATCAGTGGCGTCGTG |
| *Sirt1* | Fw | TGGCTTAGAAGATGAGCCTGA |
|  | Rv | TCCATCAGTCCCAAATCCAG |
| *TGFBI* | Fw | CGAGTGCTGTCCTGGATATG |
|  | Rv | CCCAGGGTCTCGTAAAGGTT |
| *TIMP3* | Fw | CACCCCTCACCTGTGGAA |
|  | Rv | TGACCCAAACCAGAACCAAC |
| *TNF-α* | Fw | CAGCCTCTTCTCCTTCCTGA |
|  | Rv | GCCAGAGGGCTGATTAGAGA |
| *TP53* | Fw | TGTTCTTGCAGTTAAGGGTTAGTTT |
|  | Rv | TGAAGTGGGCCCCTACCTA |
| *HPRT1* | Fw | TGACCTTGATTTATTTTGCATACC |
|  | Rv | CGAGCAAGACGTTCAGTCCT |
| *PPIA* | Fw | ATGCTGGACCCAACACAAAT |
|  | Rv | TCTTTCACTTTGCCAAACACC |
